# Biogeographic traits of dimethyl sulfide and dimethylsulfoniopropionate cycling in polar oceans

**DOI:** 10.1186/s40168-021-01153-3

**Published:** 2021-10-16

**Authors:** Zhao-Jie Teng, Qi-Long Qin, Weipeng Zhang, Jian Li, Hui-Hui Fu, Peng Wang, Musheng Lan, Guangfu Lu, Jianfeng He, Andrew McMinn, Min Wang, Xiu-Lan Chen, Yu-Zhong Zhang, Yin Chen, Chun-Yang Li

**Affiliations:** 1 State Key Laboratory of Microbial Technology, Marine Biotechnology Research Center, Shandong University, Qingdao, 266237 China; 2 College of Marine Life Sciences, Institute for Advanced Ocean Study, Ocean University of China, Qingdao, 266003 China; 3 Laboratory for Marine Biology and Biotechnology, Pilot National Laboratory for Marine Science and Technology (Qingdao), Qingdao, 266373 China; 4 The Key Laboratory for Polar Science MNR, Polar Research Institute of China, Shanghai, 200136 China; 5 Institute for Marine and Antarctic Studies, University of Tasmania, Hobart, Tasmania Australia; 6 School of Life Sciences, University of Warwick, Coventry, UK

**Keywords:** Polar oceans, DMS/DMSP cycling, Geographic distribution, Phylogenetic diversity

## Abstract

**Background:**

Dimethyl sulfide (DMS) is the dominant volatile organic sulfur in global oceans. The predominant source of oceanic DMS is the cleavage of dimethylsulfoniopropionate (DMSP), which can be produced by marine bacteria and phytoplankton. Polar oceans, which represent about one fifth of Earth’s surface, contribute significantly to the global oceanic DMS sea-air flux. However, a global overview of DMS and DMSP cycling in polar oceans is still lacking and the key genes and the microbial assemblages involved in DMSP/DMS transformation remain to be fully unveiled.

**Results:**

Here, we systematically investigated the biogeographic traits of 16 key microbial enzymes involved in DMS/DMSP cycling in 60 metagenomic samples from polar waters, together with 174 metagenome and 151 metatranscriptomes from non-polar *Tara* Ocean dataset. Our analyses suggest that intense DMS/DMSP cycling occurs in the polar oceans. DMSP demethylase (DmdA), DMSP lyases (DddD, DddP, and DddK), and trimethylamine monooxygenase (Tmm, which oxidizes DMS to dimethylsulfoxide) were the most prevalent bacterial genes involved in global DMS/DMSP cycling. Alphaproteobacteria (Pelagibacterales) and Gammaproteobacteria appear to play prominent roles in DMS/DMSP cycling in polar oceans. The phenomenon that multiple DMS/DMSP cycling genes co-occurred in the same bacterial genome was also observed in metagenome assembled genomes (MAGs) from polar oceans. The microbial assemblages from the polar oceans were significantly correlated with water depth rather than geographic distance, suggesting the differences of habitats between surface and deep waters rather than dispersal limitation are the key factors shaping microbial assemblages involved in DMS/DMSP cycling in polar oceans.

**Conclusions:**

Overall, this study provides a global overview of the biogeographic traits of known bacterial genes involved in DMS/DMSP cycling from the Arctic and Antarctic oceans, laying a solid foundation for further studies of DMS/DMSP cycling in polar ocean microbiome at the enzymatic, metabolic, and processual levels.

Video Abstract

**Supplementary Information:**

The online version contains supplementary material available at 10.1186/s40168-021-01153-3.

## Introduction

The volatile organosulfur compound dimethyl sulfide (DMS) is the main source of marine sulfate aerosols [1], a key player in the global sulfur cycle [2], and an important nutrient for many organisms (e.g., marine algae [3], coral reefs [4], and heterotrophic bacteria [5]). Although DMS can be produced and removed by a variety of abiotic processes, biological transformations, particularly bacterial production and consumption, exert great influence on the oceanic DMS budget [6].

**Fig. 1:**
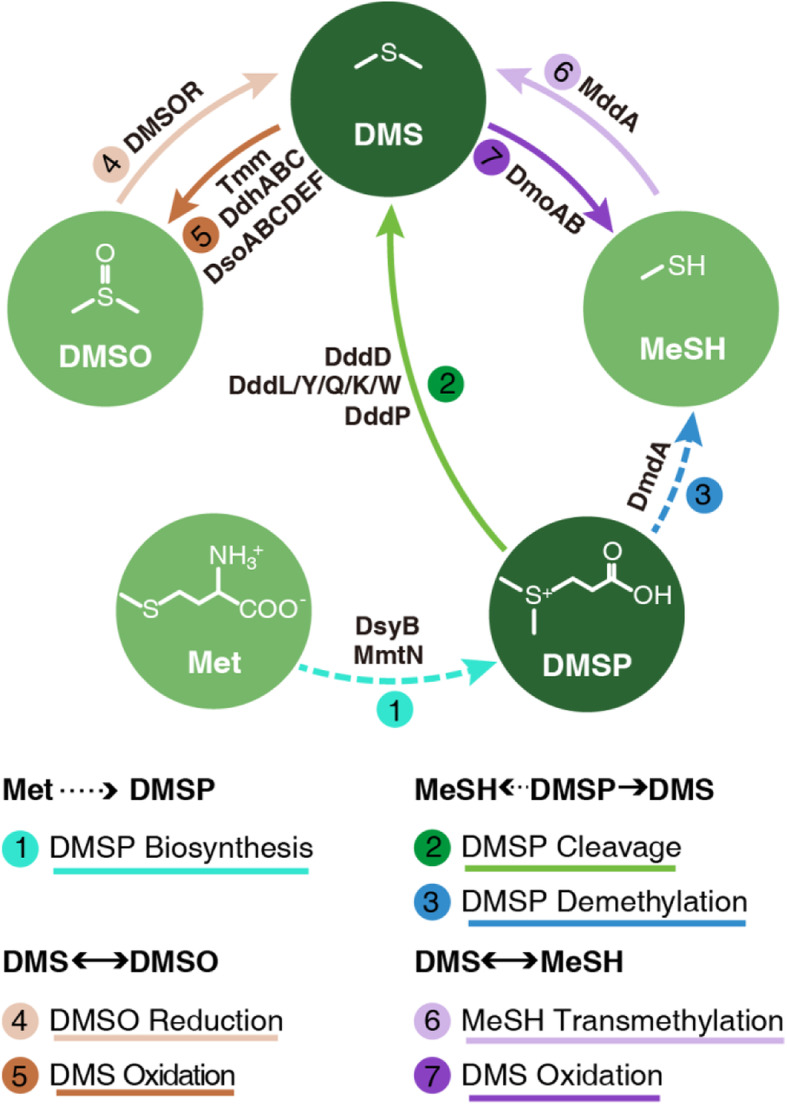
The conceptual sketch of known key proteins and pathways involved in microbial DMS/DMSP cycling. The different catabolic pathways are marked in different colours and distinguished using numbers 1–7. The dotted arrow indicated that a series of enzymatic reactions are required to form the end product

The predominant source of oceanic DMS is bacterial cleavage of dimethylsulfoniopropionate (DMSP). DMS can also be produced by direct cleavage of intracellular DMSP in DMSP-producing phytoplankton [7]. DMSP cleavage is mediated via several known DMSP lyases, including an algal DMSP lyase (Alma1) [7] and 7 bacterial DMSP lyases (DddD, DddL, DddY, DddQ, DddK, DddW, and DddP) (Table 1, Fig. 1) [6]. Dimethylsulfoxide (DMSO) is another precursor of DMS, which is ubiquitous in surface ocean waters, the sea-ice zone and sediments [25–27]. DMSO can be reduced to DMS in marine algae although the enzymes involved remain to be identified [3]. In bacteria, DMSO reduction to DMS is carried out by the DMSO reductase, DMSOR (Fig. 1) [28]. Moreover, DMS production can also be mediated by the microbial transmethylation of methanethiol (MeSH) via a methyltransferase MddA (Fig. 1) [24]. Similar to DMS, MeSH is also a volatile organic sulfur compound [29]. The transformation of MeSH to DMS plays a role in DMS production in both marine and terrestrial environments [24, 30].

**Table 1:** A list of key enzymes involved in DMS/DMSP cycling

Substrate	End product	Key enzyme	Function	Pathway	Polypeptide class	Ref
Met	DMSP	DSYB	MTHB methyltransferase	DMSP biosynthesis	SAM-dependent methyltransferase, Pfam family (PF10672)	[8]
		TpMMT	MTHB methyltransferase		SAM-dependent methyltransferase, Pfam family (PF10672)	[9]
		**DsyB**	MTHB methyltransferase		SAM-dependent methyltransferase, Pfam family (PF10672)	[10]
		**MmtN**	Met methyltransferase		Class I SAM-dependent methyltransferase family (PF10672)	[11]
DMSP	DMS	Alma1	DMSP lyase	DMSP cleavage	Aspartate racemase superfamily	[7]
		**DddD**			Class III acetyl CoA-transferase family	[12]
		**DddL**			Cupin family	[13]
		**DddY**				[14]
		**DddQ**				[15]
		**DddK**				[16]
		**DddW**				[17]
		**DddP**			M24B metallopeptidase family	[18]
	MeSH	**DmdA**	DMSP demethylase	DMSP demethylation	Glycine cleavage system T family	[19]
DMSO	DMS	**DMSOR**	DMSO reductase	DMSO reduction	DMSO reductase family	[3]
DMS	DMSO	**Tmm**	TMA monooxygenase	DMS oxidation	Class B flavoprotein monooxygenases	[20]
		**DsoABCDEF**	Monooxygenase		Phenol hydroxylase subunit super family	[21]
		**DdhABC**	DMS dehydrogenase		DMSO reductase family	[22]
	MeSH	**DmoAB**	DMS monooxygenase	DMS oxidation	Flavin-linked monooxygenases, luciferase family	[23]
MeSH	DMS	**MddA**	SAM-dependent methyltransferase	MeSH transmethylation	SAM-dependent methyltransferase, Pfam family (PF10672)	[24]

Enzymes originated from eukaryotes are shown in regular text and those from bacteria are highlighted in bold. *MTHB* methylthiohydroxybutryate, *Met* methionine, *DMSP* dimethylsulfoniopropionate, *DMSO* dimethyl sulfoxide, *TMA* trimethylamine, *SAM S*-adenosyl methionine

Bacterial oxidation is the primary process for DMS removal in the marine environment [31]. Microbial oxidation of DMS to DMSO represents a major sink of DMS in surface seawater [32]. Three enzymes capable of DMS oxidation have been identified, the multicomponent monooxygenase DsoABCDEF [21], the DMS dehydrogenase DdhABC [22], and the flavin-containing trimethylamine (TMA) monooxygenase Tmm [20]. DMS can also be converted to MeSH by the two-component DMS monooxygenase DmoAB (Fig. 1) [23].

The biosynthesis of DMSP is initiated from methionine (Met) through four different pathways, including two methylation pathways, a transamination pathway, and a decarboxylation pathway [6]. It is generally accepted that marine phytoplankton are likely the main producers [33] although marine bacteria are also known to produce DMSP [10, 11]. To date, two eukaryotic isozymes in the key step of the transamination pathway have been identified, methylthiohydroxybutryate (MTHB) methyltransferases DSYB [8] and TpMMT [9]. In bacteria, the *dsyB* gene encoding a MTHB methyltransferase in the transamination pathway [10] and the *mmtN* gene encoding a Met methyltransferase in the methylation pathway [11] have been identified very recently (Fig. 1). As the main precursor of DMS, DMSP is the most abundant organosulfur compound in the marine environment [34]. It is estimated that DMSP synthesis accounts for ~ 1 to 10% of global marine primary production [35]. In addition to the cleavage pathway which transforms DMSP to DMS, DMSP can be also metabolized to MeSH via a demethylation pathway [35]. It is estimated that between 50 and 90% of DMSP is metabolized by marine bacteria through this pathway [36]. The first step of the demethylation pathway is conducted by the DMSP demethylase DmdA, which converts DMSP to methylmercaptopropionate (MMPA); MMPA is subsequently transformed to MeSH through a series of catalytic reactions (Fig. 1) [19, 35]. DmdA is thought to be present in up to 20% of marine bacteria, mainly in the marine *Roseobacter* and SAR11 clades [37, 38]. Most recently, a structurally unusual metabolite, dimethylsulfoxonium propionate (DMSOP), has been reported, which is produced from DMSP and a previously undescribed biogenic source of DMSO [39]. However, enzymes involved in the metabolism of DMSOP have not yet been identified.

Although historically, each pathway in DMS/DMSP cycling is discovered independent, many of these genes co-exist in bacteria. For example, it is reported that *Ruegeria pomeroyi* DSS-3 has 3 different DMSP lyases, DddQ [40], DddW [17], and DddP [18], the DMSP demethylase DmdA [35] as well as the trimethylamine monooxygenase Tmm [32]. The SAR11 clade marine bacterium *Pelagibacter* sp. HTCC1062 contains DddK [41], DmdA [42], and Tmm [20]. It is also capable of catabolizing DMSP to DMS and MeSH [43] and DMS oxidation to DMSO [32]. The DMSP-producing bacterium *Labrenzia aggregata* LZB033 possessing methyltransferase DsyB can also carry out DMSP cleavage using the DMSP lyase DddL [10]. However, the reason why one bacterium carrying different types of enzymes involved in DMS/DMSP cycling, and its ecological function remain elusive.

The Arctic and Antarctic are two of the most geographically separated bioregions on Earth with extreme environmental conditions. High concentrations of DMS/DMSP have been detected in both the Arctic Ocean and the Southern Ocean (Table 2). Indeed, the world’s highest concentration of DMS in marine surface water was recorded in the Southern Ocean, which contributes significantly to the global oceanic DMS sea-air flux [60, 61]. Previous studies on Arctic and Antarctic DMS/DMSP cycling mainly focused on quantifying the spatial and temporal concentrations as well as the turnover rates of these compounds [44, 55]. Investigations on the abundance and diversity of potential genes involved in DMS/DMSP cycling in polar oceans are limited to a few selected genes involved in DMSP degradation, e.g., DmdA, DddD, DddL, and DddP, via metagenomics [62, 63], qPCR [37], or gene clone library analyses [64, 65]. With the global warming threat, the polar regions are experiencing rapid changes including sea ice melting [66, 67] that is known to correlate with the reduced production of DMS/DMSP [61, 68], and this, in turn, may feedback to the global climate. Thus, interpreting the biogeographic traits of DMS/DMSP cycling in Arctic and Antarctic oceans is an urgent task. We postulate that the biogeographic traits of DMS/DMSP cycling in polar oceans may be similar and are less affected by dispersal limitation since similar microbial community structure was observed in these regions [69]. Moreover, considering high concentrations and fast turnover rates of DMS/DMSP have been recorded in polar oceans [26, 27], we hypothesize that genes involved in DMS/DMSP cycling are common in polar ocean microbiome. In this study, we set out to systematically uncover the distribution and abundance of 16 functional microbial enzymes involved in DMS/DMSP cycling (Table 1) in the Arctic and Antarctic oceans via metagenomic and metatranscriptomic analyses in order to gain a global overview of microbial transformation of DMS/DMSP in polar oceans.

**Table 2:** DMS and DMSP concentrations in environmental samples obtained from the polar oceans

	Type	Sampling time	DMS (nM)	DMSPt (nM)	Ref
**Arctic**					
Canadian High Arctic	Surface water	Oct to Nov 2007	(0.05–0.80)	DMSPp (2–39)	[44]
				DMSPd (< 2)	
Barents Sea	Surface water	May 1993	5.20 (2–22.50)	DMSPp (6–10)	[45]
				DMSPd (4–8)	
Baffin Bay/Lancaster Sound	Surface water	Sep 2008	1.31	DMSPp 18.20 (5–70)	[46]
				DMSPd 0.80 (0.30–2.10)	
Baffin Bay	Water column	Apr to Jun 1998	0.60 (0–6.70)	(0–9.50)	[47]
	Sea ice		-	DMSPp 126 (8.70–987)	[48]
Central Arctic Ocean	Surface water	Aug to Oct 1991	(0.04–12)	-	[49]
Storfjorden	Surface water	Aug 2005	-	DMSPp (5–50)	[50]
	Subsurface and brine enriched water		-	DMSPp (< 10)	
**Antarctic**					
Amundsen Sea	Polynya waters and sea ice zone	Jan to Feb 2009	(< 1–350)	-	[51]
Prydz Bay	Coastal waters	Dec 1988 to Feb 1989	(12–111)	-	[52]
Davis Station	Coastal waters	May 1987 to Jan 1988	(1–290)	(1–100)	[53]
Adélie Land	Platelet ice-like layer	Nov to Dec 1999	(4–74)	-	[54]
	Brines		(20–60)	-	
	Underlying water		(1–17)	-	
East Antarctica	Upper 150 m of the water column	Jan to Mar 2006	8 (0–63)	DMSPp 11 (nd-38)	[55]
				DMSPd 5 (nd-36 )	
				DMSPt 16 (nd-54)	
Ross Sea	Sub-euphotic water column	Dec 2004 to Jan 2005, Nov 2005	-	(0.5–22)	[56]
Palmer Station	Surface seawater	Jan to Feb 1994	(0.70–3.70)	-	[57]
	Coastal waters	Oct 2012 and Mar 2013	(0–20)	(8–160)	[27]
Weddell Sea	Open water	Oct to Nov 1988	-	12.20 (6.50–22.90)	[58]
	Ice zone			10.50 (0.40–46.10)	
	Brine			61.80 (7.55–203.60)	
	Pack ice			322 (4–1664)	
Drake Passage to the Bellingshausen Sea	Surface waters	Oct to Nov 1992	(0.15–27)	(2–69)	[59]
	Ice cores		2 (0.2–27)	(1–28)	

DMS/DMSP concentration are shown in mean (range). *DMSPt* total DMSP, *DMSPd* dissolved DMSP, *DMSPp* particulate DMSP

## Materials and methods

### Bioinformatic analyses of genes involved in DMS/DMSP cycling

**Fig. 2:**
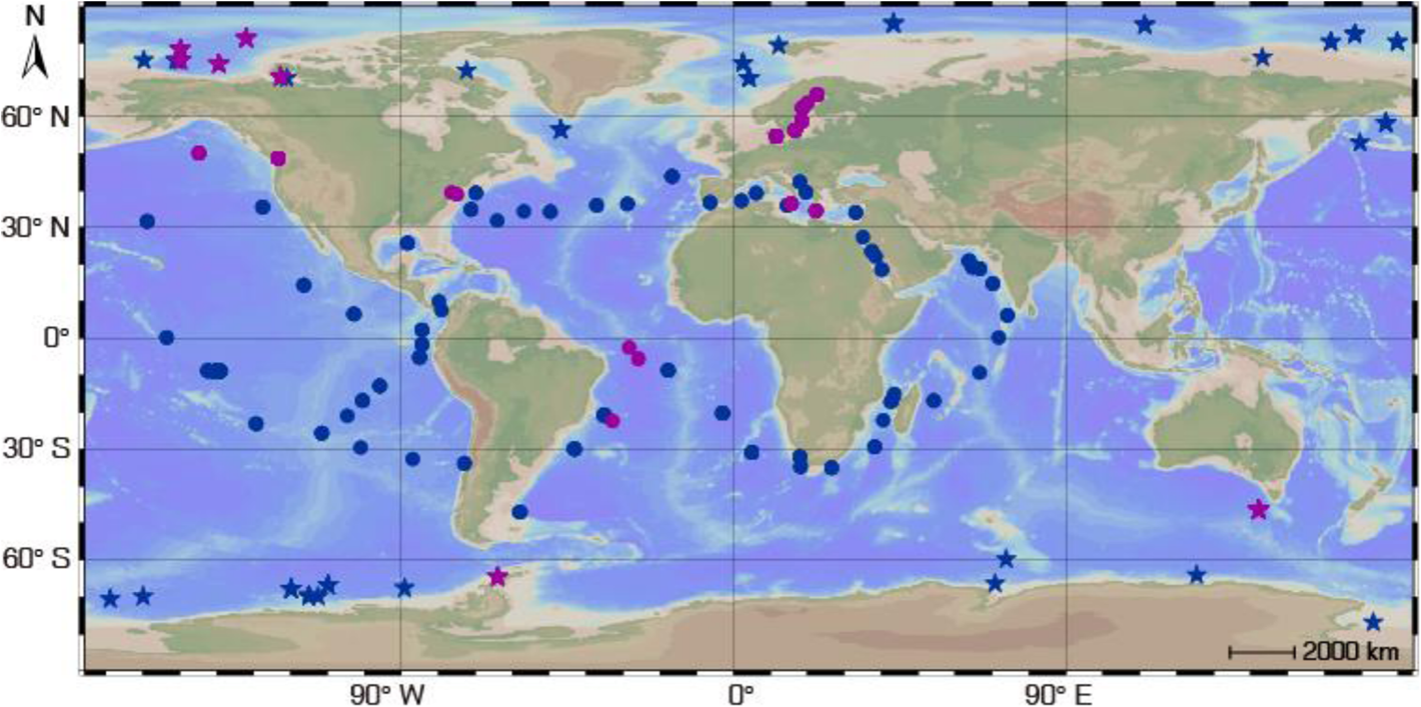
Geographic distribution of the sampling locations of the metagenomic (blue symbols) and metatranscriptomic (purple symbols) samples from polar (indicated by stars) and non-polar (indicated by dots) ocean

The sampling locations (Fig. 2), sequencing, and assembly of 60 polar seawater samples (Table S1, NCBI BioProject accession no. PRJNA588686) and 214 metagenome assembled genomes (MAGs, Table S3, NCBI BioProject accession no. SUB7116349) have been described previously [69]. These polar seawater samples were prefiltered through 20-μm polycarbonate membrane filters (Millipore, MA, USA) and cells were then filtered onto 0.22-μm polycarbonate membrane filters, as such algal genes involved in DMSP/DMS metabolism were not analysed in this study. According to the sampling locations and depths, the 60 metagenomic samples were separated into four groups: Arctic-Surface (0–100 m, *n* = 16), Arctic-Deep (300–3800 m, *n* = 23), Antarctic-Surface (0 m, *n* = 12), and Antarctic-Deep (300–3500 m, *n* = 9). For comparison, metagenomes of 174 non-polar seawater samples (Table S4) were also analysed, including 139 surface seawater (5–188 m) and 35 deep seawater (250–1000 m) samples from the *Tara* Oceans project [70] (fraction size, 0.22–3 μm; http://www.pangaea.de/). Additionally, 151 metatranscriptomes (99 non-polar seawater samples and 52 polar seawater samples; Table S5) and all microbial genomes (as of March 9, 2021) in the IMG/M database [71] were also used for analysis.

The functionally ratified protein sequences (Table 3), namely MmtN, DsyB, DddD, DddK, DddP, DddQ, DddW, DddL, DddY, DmdA, DMSOR, Tmm, DsoB (a key catalytic subunit of monooxygenase DsoABCDEF), DdhA (the catalytic subunit of DMS dehydrogenase DdhABC), MddA, and DmoA (the catalytic subunit of DMS monooxygenase DmoAB), were obtained from the National Center for Biotechnology Information (NCBI) database (https://www.ncbi.nlm.nih.gov/) or the IMG/M database [71]. Homologues of DsoB and DmoA in metagenomes/metatranscriptomes were obtained using BLASTP, since both of which only had one biochemically characterized protein (Table 3). For the other 14 proteins involved in DMS/DMSP cycling, hidden Markov models (HMM) were created for each enzyme using protein sequences that are biochemically or structurally characterized and their homologues from metagenomes/metatranscriptomes were obtained using hmmsearch (http://hmmer.org). The cutoff values used were selected based on established stringency cutoff values from previous reports (Table 3). The sequences retrieved from our bioinformatics pipeline were further scrutinized for the presence of key residues involved in substrate binding or catalysis and/or validated through protein purification and further biochemical characterization (see below). The amino acid sequences of 10 conserved bacterial marker genes [75] were retrieved from the NCBI database, and the average abundance of these marker genes was used to normalize the abundance of the genes involved in DMS/DMSP cycling in metagenomic and metatranscriptomic datasets as described previously [10].

**Table 3:** The functionally ratified protein homologues of DMS/DMSP cycling-related enzymes

Protein	Source of strain	Gene ID^**#**^	***e***-value cutoff	Identity cutoff	Method	Ref
DsyB	*Thalassobaculum salexigens* DSM 19539	2523405058	1E− 67	--	HMMsearch	[10]
	*Amorphus coralli* DSM 19760	2517908241				
	*Oceanicola batsensis* HTCC2597	638883374				
	*Sagittula stellata* E-37	640641694				
	*Sediminimonas qiaohouensis* DSM 21189	2523943366				
	*Labrenzia aggregata* LZB033	AOR83342.1				
	*Labrenzia aggregate* IAM12614	WP_075282486.1				
MmtN	*Roseovarius indicus* B108	WP_143100449.1	1E− 50	--	HMMsearch	[11]
	*Thalassospira profundimaris* WP0211	2530549224				
	*Novosphingobium* sp. MBES04	2631597816				
	*Nocardiopsis chromatogenes* YIM90109	2554031325				
	*Streptomyces mobaraensis* NBRC13819	2538966579				
DddD	*Marinomonas* sp. MWYL1	WP_012071702.1	1E− 30	--	HMMsearch	[72]
	*Sinorhizobium fredii* NGR234	AAQ87407.1				
	*Burkholderia ambifaria* AMMD	WP_011659284.1				
	*Halomonas* sp. HTNK1	ACV84065.1				
	*Pseudomonas* sp. J465	ACY01992.1				
	*Psychrobacter* sp. J466	ACY02894.1				
	*Oceanimonas doudoroffii*	AEQ39135.1				
DddL	*Sulfitobacter* sp. EE-36	ADK55772.1	1E− 30	--	HMMsearch	[72]
	*Rhodobacter sphaeroides* 2.4.1	Q3J6L0.1				
	*Thioclava pacifica*	WP_051692700.1				
	*Puniceibacterium antarcticum* SM1211	WP_099909581.1				
DddY	*Alcaligenes faecalis* M3A	WP_123051132.1	1E− 30	--	HMMsearch	[72]
	*Shewanella chilikensis*	PYE57415.1				
	*Acinetobacter bereziniae* NIPH 3	5Y4K_A				
DddQ	*Ruegeria pomeroyi* DSS-3	Q5LT18.1	1E− 30	--	HMMsearch	[72]
	*Ruegeria lacuscaerulensis* ITI-1157	SHI35160.1				
DddK	*Pelagibacter ubique* HTCC1062	WP_011281678.1	1E− 30	--	HMMsearch	this study
	Pelagibacteraceae bacterium BACL20 MAG-120920-bin64	KRP06000.1				
	Alpha proteobacterium HIMB5	AFS47241.1				
	*Candidatus* Pelagibacter ubique	WP_006997514.1				
DddW	*Ruegeria pomeroyi* DSS-3	WP_011046214.1	1E− 30	--	HMMsearch	[72]
	*Roseobacter* sp. MED193	EAQ44306.1				
DddP	*Roseovarius nubinhibens* ISM	A3SK19.1	1E− 30	--	HMMsearch	[72]
	*Ruegeria pomeroyi* DSS-3	AAV95561.1				
	*Phaeobacter inhibens* DSM 17395	AFO91571.1				
	*Ruegeria lacuscaerulensis* ITI-1157	SHJ09750.1				
	*Oceanimonas doudoroffii* DSM 7028	AEQ39091.1				
	*Oceanimonas doudoroffii* DSM 7028	AEQ39103.1				
	*Aspergillus oryzae* RIB40	BAE62778.1				
DmdA	*Ruegeria pomeroyi* DSS-3	AAV95190.1	1E− 50	--	HMMsearch	[73]
	*Pelagibacter ubique* HTCC1062	Q4FP21.1				
	*Dinoroseobacter shibae* DFL 12	WP_012178987.1				
	*Marine gammaproteobacterium* HTCC2080	WP_007233625.1				
	*Granulosicoccus antarcticus* IMCC3135	ASJ73090.1				
DMSOR	*Rhodobacter sphaeroides* f. sp. *denitrificans*	BAA07615.1	1E− 50	--	HMMsearch	this study
	*Rhodobacter capsulatus*	Q52675.2				
Tmm	*Roseovarius* sp. 217	EAQ26624.1	1E− 80	--	HMMsearch	[20]
	*Ruegeria pomeroyi* DSS-3	AAV94838.1				
	*Methylophaga* sp. SK1	JC7986				
	*Methylocella silvestris* BL2	ACK52489				
	*Pelagibacter ubique* HTCC1002	EAS85405.1				
	*Pelagibacter ubique* HTCC7211	EDZ59919.1				
DsoB	*Acinetobacter guillouiae* strain 20B	BAA23331.1	1E− 30	≥ 40	Blastp	this study
DdhA	*Sagittula stellata* E-37	EBA07058.1	1E− 50	--	HMMsearch	this study
	*Rhodovulum sulfidophilum* strain SH1	AAN46632.1				
DmoA	*Hyphomicrobium sulfonivorans*	E9JFX9.1	1E− 30	≥ 40	Blastp	[23]
MddA	*Pseudomonas deceptionensis* M1	AJE75769.1	1E− 30	--	HMMsearch	[72]
	*Cyanothece* sp. ATCC 51142	WP_009545670.1				
	*Mycobacterium tuberculosis* H37Rv	NP_217755.1				
	*Pseudomonas* sp. GM41	WP_008148420.1				
	*Bradyrhizobium diazoefficiens* USDA 110 (Blr1218)	WP_011084036.1				
	*Bradyrhizobium diazoefficiens* USDA 110 (Blr5741)	WP_011088485.1				
MTO	*Hyphomicrobium* sp. VS	ATJ26742.1	1E− 20	--	HMMsearch	[74]
	*Ruegeria pomeroyi* DSS-3	WP_011242048.1				
	*Hyphomicrobium denitrificans* ATCC 51888	ADJ22562.1				
	*Pseudovibrio ascidiaceicola* DSM 16392	WP_093522951.1				
	*Methylococcus capsulatus* str. Bath	AAU90430.1				

^#^Gene ID in either the IMG/M database or the GenBank database

### Curation and validation of predicted DMS/DMSP cycling-related genes

To further validate the environmental sequences retrieved from these marine metagenomes/metatranscriptomes, several approaches were applied to curate these datasets. Firstly, all hits of the top 5 most abundant enzymes (DddD, DddP, DddK, DmdA, and Tmm) were retrieved from the metagenomes/metatranscriptomes and aligned by MUSCLE. Maximum-likelihood phylogenetic trees were created via FastTree [76] and visualized through EvolView [77]. Phytogenic affiliation of the predicted hits was assessed using other enzymes of the same protein family as outgroups (Table S2).

Second, for those enzymes involved in DMSP/DMS cycling whose structures are available (i.e., DddK [41], DddQ [40], DddY [78], Tmm [79], and DddP [80], DMSOR [81], and DmdA [82]), we performed multiple sequence alignment of environmental hits using MUSCLE [83] and analysed the conserved key resides involved in substrate-coordination and catalysis (Figure S1).

Finally, to validate the function of predicted hits of the top 5 most abundant enzymes from our datasets, we randomly selected several environmental sequences from each group, chemically synthesized these genes, and overexpressed them in recombinant *Escherichia coli* for functional characterization of their enzyme activities. These included DddD (2 sequences), DddP (2 sequences), DddK (2 sequences), DmdA (1 sequence), and Tmm (2 sequences) (Table S6). The nucleotide sequences of these 9 hits were synthesized by BGI (Beijing, China), cloned, and overexpressed using the pET22b plasmid in *Escherichia coli* BL21 (DE3). These proteins were purification as described previously [84] and their activities were measured following the protocols from previous reports (Table S6) [12, 32, 41, 72, 80]. The newly identified DddX was not analysed in this study [85]. DddX homologs returned from *Tara* Oceans datasets are usually short and do not always contain the full open reading frame, making it difficult for gene synthesis and overexpression in *E. coli* for functional validation.

### Taxonomic profiling

The amino acid sequences of predicted DMS/DMSP cycling-related genes from these metagenomes/metatranscriptomes were extracted using scripts compiled in Python code and aligned against the non-redundant protein sequences (nr) database using BLASTP [86]. The best hit of each query sequence was retrieved, and its taxon was recorded. Taxonomic classification of the assembled MAGs was performed with GTDB-Tk v0.3.2 (the script classify wf was used) [87] using the Genome Taxonomy Database (GTDB) [88].

### Data analysis and visualization via bioinformatics tools

The geographical distribution of sampling locations was constructed by Ocean Data View [89]. DMS/DMSP-related protein homologs retrieved from these marine metagenomes/metatranscriptomes were analysed and visualized using the R software package [90] with the following descriptions. Relative abundance and phylogenetic diversities of DMS/DMSP cycling-related genes in polar metagenomic samples were visualized using the ‘gplots’ and the ‘ggplot2’ package [91], respectively. The Sankey diagram of the taxonomic profiling of DMS/DMSP cycling-related genes was built using the ‘ggalluvial’ package [92]. For principal coordinates analysis (PCoA), gross relative abundance in each metagenomic sample was normalized to 1, and Bray-Curtis distances were generated using the ‘vegan’ packages [93], based on the percentages of DMS/DMSP-related genes. Redundancy analysis (RDA) was performed based on the relative abundance of DMS/DMSP-related genes using the ‘vegan’ package. Geographical distance was generated using the ‘geosphere’ package (https://cran.r-project.org/web/packages/geosphere/index.html). The relationship between Bray-Curtis dissimilarity of microbial communities [94] involved in DMSP/DMS cycling and geographic distance or water depth were analysed using the Mantel test. Alpha-diversity analysis was performed on polar microbiota involved in DMS/DMSP cycling. Shannon and Simpson index was calculated using the ‘vegan’ package and plotted via Origin 2018 (https://www.originlab.com/). The average abundance of DMS/DMSP-related genes in metagenomic and metatranscriptomic samples from polar and non-polar oceans was used for Pearson correlation analysis. Pearson correlation coefficients and *P* values were calculated using ‘ggcorrplot’ packages [91]. Data processing was performed via scripts compiled in Python code. All graphs were combined via Adobe Illustrator CS5.

## Results

### Curation of the environmental sequences obtained from polar and non-polar oceans and abundance of genes involved in DMS/DMSP cycling

**Fig. 3:**
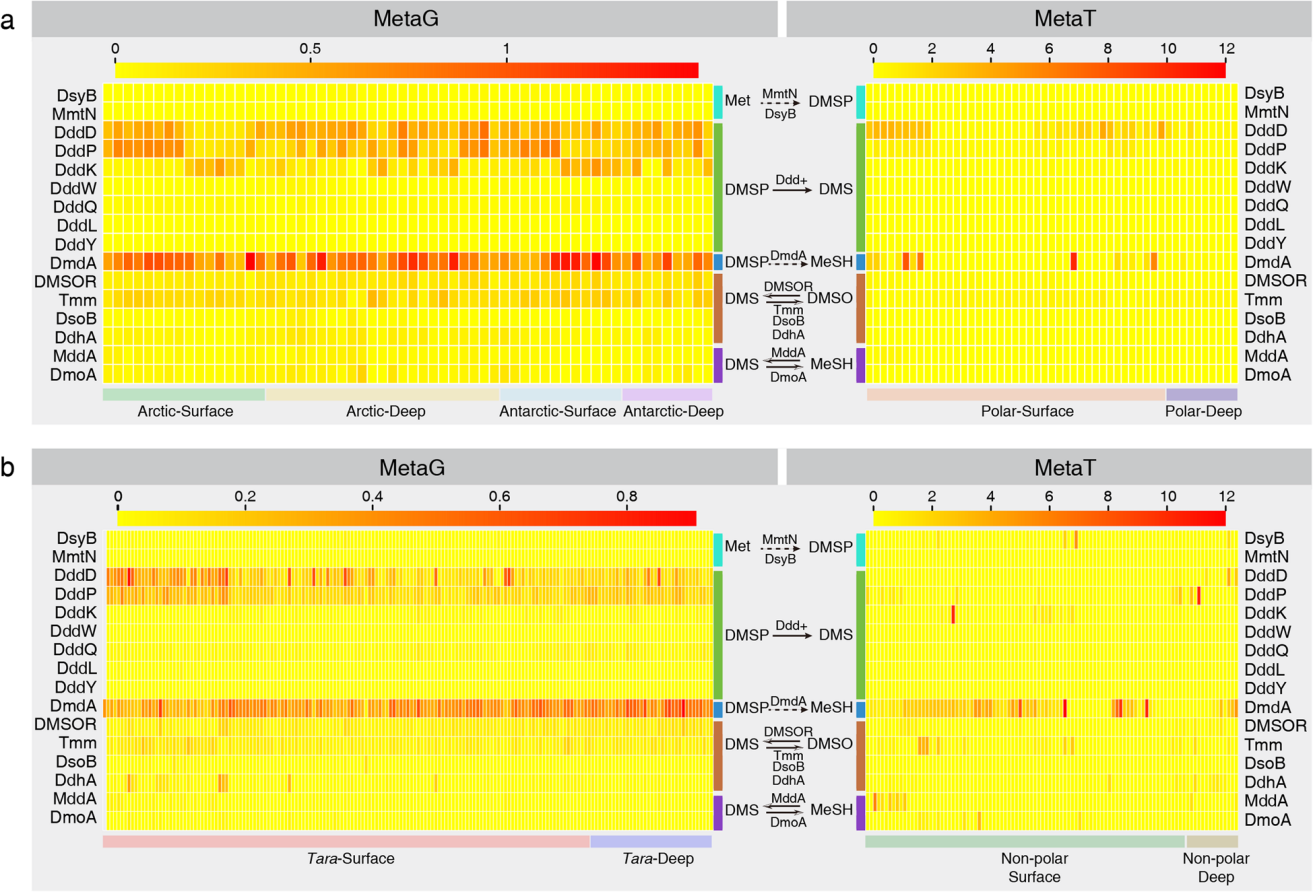
Relative abundance of potential genes involved in bacterial DMS/DMSP cycling. **a** The relative abundances of DMS/DMSP cycling-related genes in 60 polar metagenomes (left panel) and 52 polar metatranscriptomes (right panel). **b** The relative abundances of DMS/DMSP cycling-related genes in 174 non-polar *Tara* Ocean samples (left panel) and 99 non-polar metatranscriptomes (right panel). Polar metagenomes were separated into Arctic-Surface (0–100 m), Arctic-Deep (300–3800 m), Antarctic-Surface (0 m), and Antarctic-Deep (300–3500 m). Polar metatranscriptomic samples were separated into two groups: Polar-surface (0–146 m) and Polar-deep (200–1000 m) groups. *Tara* metagenomes and metatranscriptomes were separated into *Tara*-Surface (5–188 m) and *Tara*-Deep (250–1000 m), and Non-polar surface (0–150 m) and Non-polar deep (200–3262 m), respectively. The relative abundance of each gene was normalized against the average abundance of the 10 selected bacterial marker genes. MetaG, metagenomes; MetaT, metatranscriptomes

Wherever feasible, we built hidden Markov models (HMM) for each protein involved in DMSP/DMS cycling using ratified sequences obtained from literature (Table 1). These HMM models were then used to search the polar metagenomes and metagenomes/metatranscriptomes from the *Tara* Ocean datasets (Table 3). Homologs of all currently known bacterial enzymes in DMS/DMSP cycling (Table 1) were found in the Arctic and Antarctic seawater samples (Fig. 3a) although majority of the samples were dominated by five putative enzymes, i.e., DddD, DddP, DddK, DmdA, and Tmm. Most of these putative enzymes involved in DMSP/DMS cycling exhibited wide geographical distributions, several of which (e.g., DddD, DddP, DmdA, Tmm) were detected in all 60 polar ocean samples (Fig. 3a, Table S1).

To evaluate the validity of our approach, we used three complementary methods to curate these sequences. First, we analysed predicted hits for the occurrence of conserved amino acid resides involved in substrate coordination and catalysis guided by biochemical data and/or available protein structures (Figure S1). Our analyses suggest that the HMM model can successfully retrieve environmental sequences that largely retained the conserved sites necessary for performing corresponding enzyme activity (Figure S1). This is supported by further phylogenetic analyses performed for the top five most abundant genes in our datasets (i.e., DddD, DddP, DddK, DmdA, and Tmm), showing that the majority of the predicted hits are affiliated with ratified enzymes (Figure S2). To validate the function of these predicted proteins, we then randomly selected 9 environmental sequences from the aforementioned five protein groups and tested their corresponding enzyme activities using purified proteins from recombinant *E. coli*. Indeed, these proteins retrieved from environmental samples were functional (Table S6). Taken together, our approach appears capable of retrieving bona fide sequences involved in DMS/DMSP cycling from these polar and no-polar marine omics datasets.

In contrast to proteins involved in DMSP catabolism, bacterial DMSP biosynthesis pathway (e.g., *dsyB, mmtN*) did not appear to be prevalent in these polar samples (Table S1). In contrast, the DmdA-mediated DMSP demethylation pathway was more prevalent, consistent with previous reports of high abundance of DmdA from other oceans [37, 62, 95]. The DMSP cleavage pathway was also numerically abundant in polar oceans, and DddD, DddP, and DddK were more frequently observed than DddW/DddQ/DddL/DddY. Moreover, the potential genes involved in the transformation between DMS and DMSO were more abundant than those between DMS and MeSH (Fig. 3a). To compare the geographic distribution of DMS/DMSP cycling between the polar and non-polar oceans, the 174 non-polar metagenome samples and the 151 metatranscriptome samples from the *Tara* Oceans project were analysed. Among the 16 proteins analysed, DmdA, DddD, and DddP were also the most abundant genes involved in DMS/DMSP cycling in non-polar metagenomic samples (Fig. 3b). DMS/DMSP cycling in non-polar oceans appears to be primarily driven by the DMSP demethylation pathway (DmdA), and DddD and DddP mediated DMSP cleavage pathways (Fig. 3b). In addition, the relative abundance of potential transcripts involved in DMS/DMSP cycling in non-polar and polar metatranscriptomic samples were significantly correlated with the relative abundance of potential genes in non-polar (Pearson correlation coefficient = 0.84, *P* value < 0.0001) and polar metagenomic samples (Pearson correlation coefficient = 0.94, *P* value < 0.0001), respectively (Fig. 3).

### Geographic distribution traits of DMS/DMSP cycling in polar and non-polar oceans

**Fig. 4:**
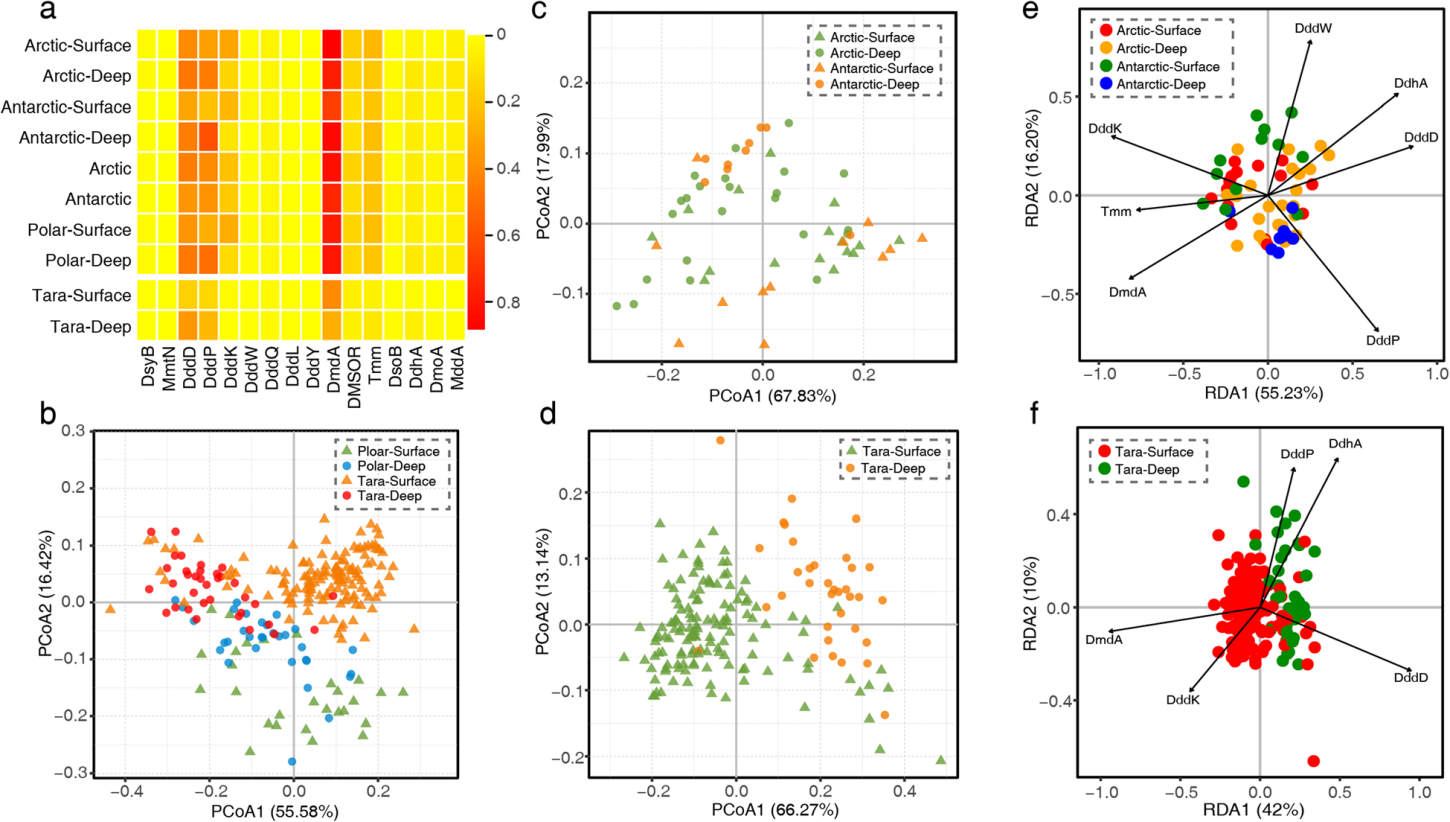
Analyses of inter-sample similarity among the polar and non-polar seawater samples. **a** Average relative abundance of DMS/DMSP-related genes in different metagenomic sample groups. Bray-Curtis dissimilarities of all (**b**), polar (**c**), and *Tara* (**d**) metagenomic samples illustrated by PCoA analysis based on the relative abundances of DMS/DMSP-related genes. The total abundance of each metagenomic sample was normalized to 1. The percentages of variation explained by the principal coordinates are indicated on the axes. RDA analyses of sampling sites and protein types of polar samples (**e**) and *Tara* samples (**f**). The ordination plot was constructed using the relative abundance of DMS/DMSP-related proteins. Proteins involved in DMS/DMSP cycling are indicated by black arrows. The percentages of variation are shown on the axes

In the metagenomic samples from the Arctic Ocean, the average relative abundance of DMS/DMSP cycling-related genes in surface waters was higher than that in deep waters. However, the opposite appears to hold true in the Southern Ocean metagenomic samples (Fig. 4a, Table S1), which may be explained by the so-called ‘high nutrient, low chlorophyll’ paradox likely caused by iron limitation in the surface layer of the Southern Ocean [96, 97]. In addition, it is noticeable that a high relative abundance of DMS/DMSP cycling-related genes, especially DMSP lyases, was found in deep seawaters over 3000 m (Fig. 4a, Table S1), implying an important role of DMS/DMSP cycling in deep ocean sulfur cycle.

To determine the distribution characteristics of DMS/DMSP-related genes in polar and non-polar oceans, principal coordinates analysis (PCoA) and redundancy analysis (RDA) were performed. These metagenomic samples were broadly grouped into three independent coordinates: polar surface waters, Tara surface waters, and deep waters (Fig. 4b). Polar and non-polar surface waters were less similar from their gene abundance. In contrast, deep waters in polar and non-polar oceans were more similar and displayed different distribution patterns compared with the surface waters (Fig. 4c, d). Hence, the distributions of DMS/DMSP-related genes were clustered primarily based on water depth rather than geographic distance.

Further RDA analysis demonstrated that the divergence of the ordinations is mostly driven by the differences of relative abundance of certain genes in DMS/DMSP cycling in surface and deep waters (Table S7). DddK was relatively more prevalent in polar surface waters, while DddD and DddP were more common in polar deep waters (Fig. 4a, e). In non-polar oceans, DmdA and DddK were the principal elements that influenced the distribution traits of surface DMS/DMSP cycling, whereas DddD and DdhA were more influential in deep waters (Fig. 4f). The high relative abundance (Fig. 4a) and wide distribution (Fig. 4e, f) of DddK in surface waters were consistent with the fact that it is primarily originated from the SAR11 clade (Pelagibacterales) which is numerically dominant in the surface ocean [43, 95], and the broad dispersion of DddD in deep waters suggests its importance in DMS/DMSP cycling in deep waters.

### Phylogenetic diversity of DMS/DMSP cycling-related genes in polar oceans

**Fig. 5:**
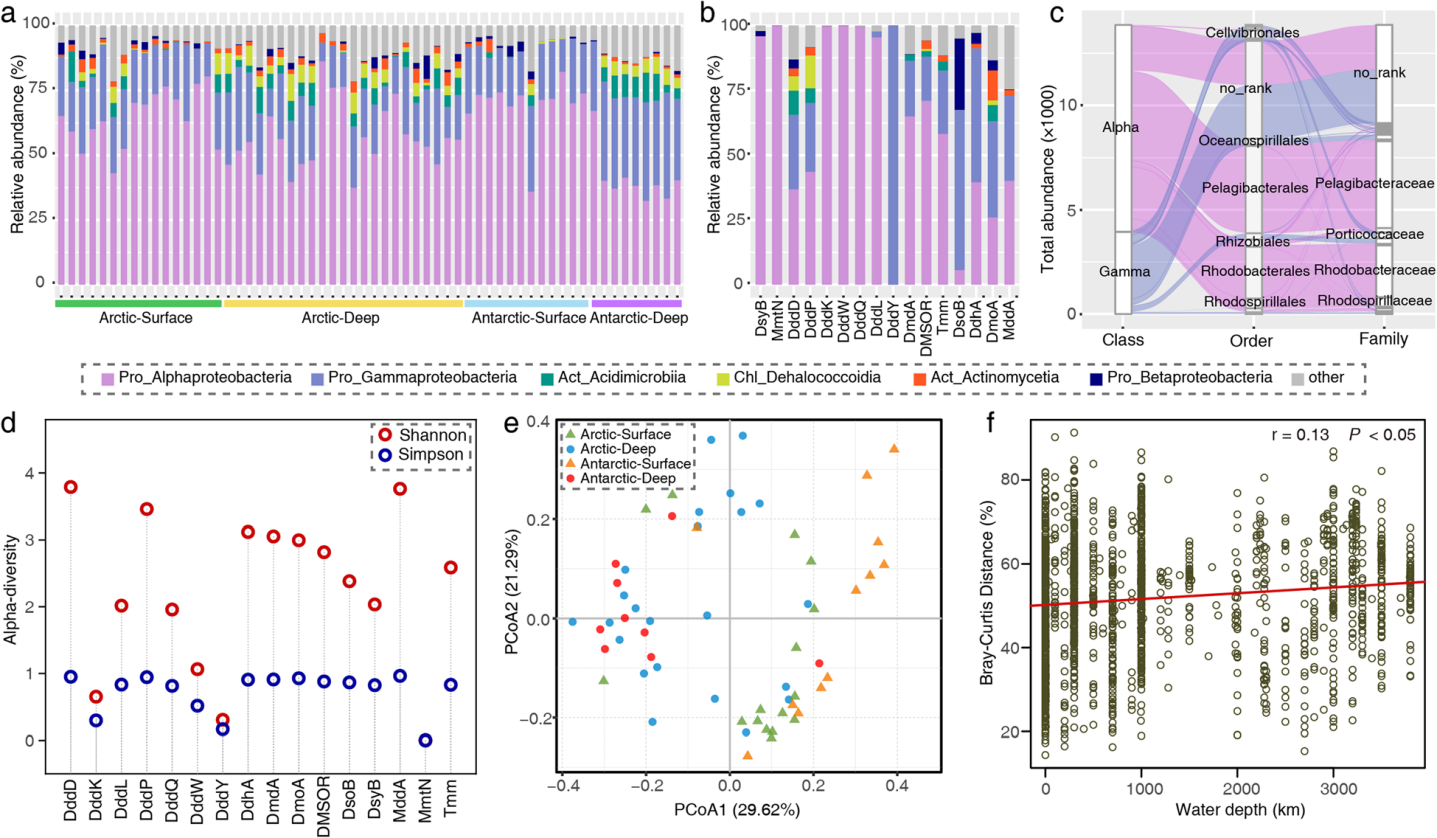
Phylogenetic diversity of DMS/DMSP cycling-related genes in the Arctic and Antarctic oceans. The taxonomic compositions of microbiota involved in DMS/DMSP cycling are displayed at the class level for sample groups (**a**) and proteins (**b**). **c** Taxonomic profiling of DMS/DMSP cycling-related microbiota in Alphaproteobacteria (Alpha) and Gammaproteobacteria (Gamma) classes. Pro, Proteobacteria; Act, Actinobacteria; Chl, Chloroflexi. **d** Alpha-diversity analyses of the polar microbiomes involved in DMS/DMSP cycling. Shannon and Simpson diversity was calculated based on the taxonomic composition of DMS/DMSP-related genes, with higher values representing higher biodiversity. **e** Bray-Curtis dissimilarities of polar metagenomic samples illustrated by PCoA analysis based on the taxonomic compositions of DMS/DMSP-related genes. The DMS/DMSP-related community composition of each metagenomic sample was normalized to 1. **f** Correlation between dissimilarity of DMS/DMSP-related bacterial community and water depth in polar oceans. The Bray-Curtis dissimilarity index was used. The correlation coefficients (*r*) and Spearman’s correlation *P* value (*P*) were indicated

To reveal the taxonomic diversity of DMS/DMSP cycling-related proteins in polar oceans, 17,189 protein sequences from polar oceans obtained through our pipeline were aligned against the NCBI-nr database, and the taxon of each best hit with the highest accuracy to species level was extracted. Thirty phyla (26 phyla from Bacteria domain, 2 phyla from Eukaryota domain and 2 phyla from Archaea domain) spanning over 38 classes, 72 orders, and 107 families were involved in polar DMS/DMSP cycling (Table S8). Among the phyla affiliated to Bacteria, Proteobacteria accounted for 84% of the total sequences, of which the dominant classes were Alphaproteobacteria (58%) and Gammaproteobacteria (23%) (Fig. 5a, b). Sequences of DddY, DsoB, DdhA, and DmoA were dominated by Gammaproteobacteria whereas the other 12 proteins were mainly affiliated with Alphaproteobacteria (Fig. 5b). In Alphaproteobacteria (9917 sequences), the Pelagibacterales (5016 sequences) were the most abundant (Fig. 5c), in which members of DmdA, DddK, and Tmm made great contributions. Indeed, Alphaproteobacteria participated in all 7 DMS/DMSP cycling pathways (Fig. 5b), in which Pelagibacterales were involved in 5 pathways (i.e., DsyB, DddD/DddK/DddP/DddQ, DmdA, DMSOR, and Tmm) indicating their role as generalists in DMS/DMSP cycling.

Regardless of the abundance of the potential genes, DddD, DddP, and MddA exhibited high phylogenetic diversities (Fig. 5d). In contrast, MmtN (100% from Sphingomonadales), DddW (100% from Rhodobacterales), DddY (100% from Alteromonadales), and DddK (99% from Pelagibacterales) were highly conserved at the order level (Table S8). Similarly, the biogeographic patterns of DMS/DMSP cycling in polar oceans were mainly driven by water depth (Fig. 5e) rather than geographical distance. In addition, the dissimilarity of community composition of DMS/DMSP-related genes among polar seawater samples was in line with a depth-decay relationship (Fig. 5f) instead of a distance-decay relationship (Figure S3). Thus, environmental conditions were likely more important than dispersal limitation in determining community composition of DMS/DMSP-related genes.

### DMS/DMSP cycling traits in MAGs obtained from polar oceans

**Fig. 6:**
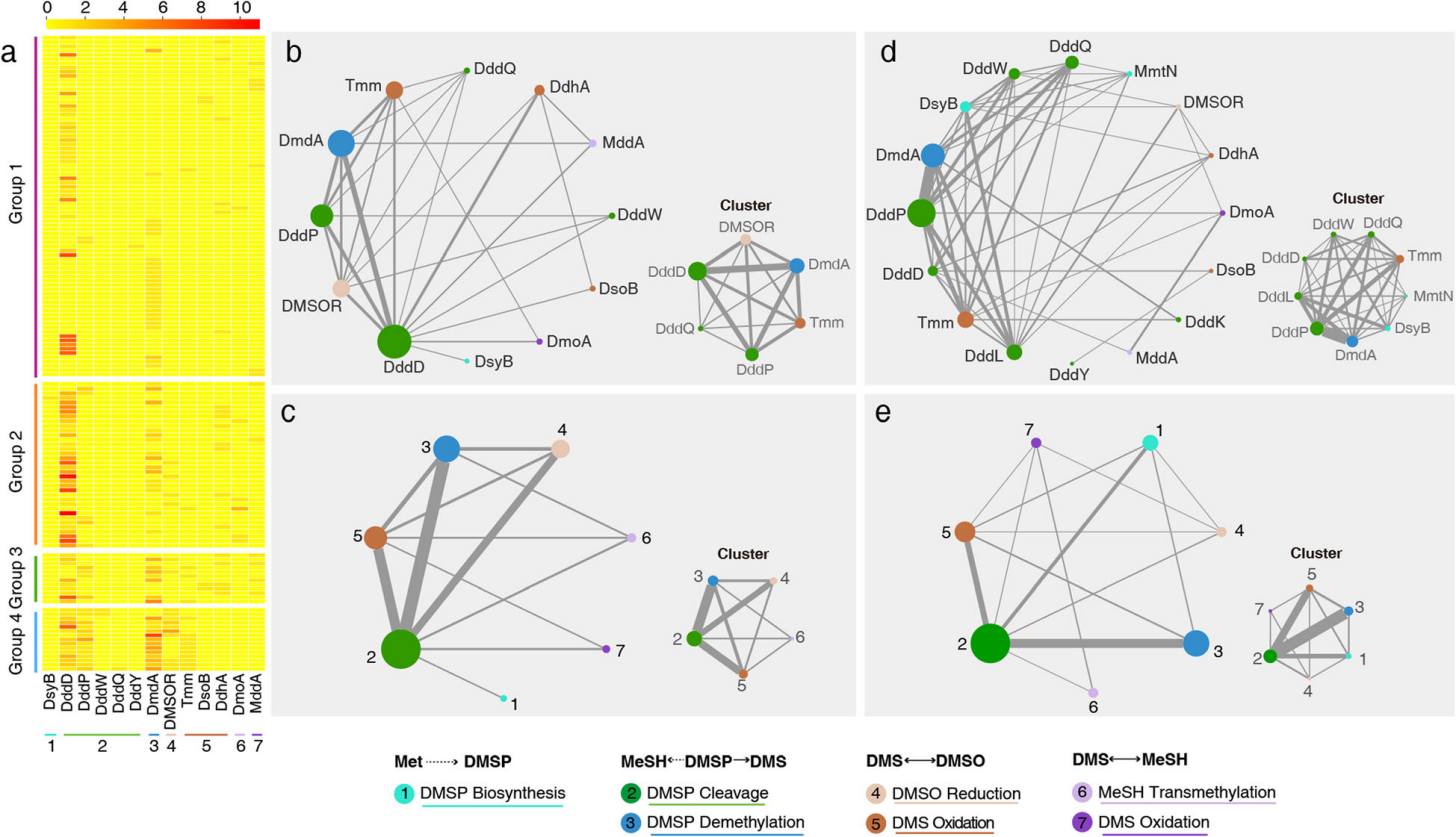
The gene frequency and taxonomic composition of polar metagenome assembled genomes (MAGs) involved in DMS/DMSP cycling in polar oceans. **a** Frequency of DMS/DMSP cycling-related genes in 143 (out of 214) polar MAGs. MAGs are separated into four groups with MAGs in groups 1 to 3 carrying 1 to 3 types of DMS/DMSP cycling-related genes, MAGs in group 4 contains more than 3 types of DMS/DMSP cycling-related genes. The co-occurrence networks of protein-protein (**b, d**) and pathway-pathway (**c, e**) coexistence modes in DMS/DMSP cycling in MAGs obtained from polar oceans (**b**, **c**) compared to all microbial genomes in the IMG/M database (**d, e**). The cluster of each network was shown in its lower left corner. Each node in the networks indicates one protein (**b**, **d**) or one pathway (**c**, **e**) involved in DMS/DMSP cycling. The proteins in the same catabolic pathway (as indicated in Table 1) are marked using the same colour, and different pathways are distinguished using numbers 1–7. The size of nodes and the thickness of edges represent the frequencies of genes and MAGs carrying multiple genes involved in DMS/DMSP cycling, respectively.

In the majority of the metagenomic samples from both polar and non-polar oceans, the cumulative relative abundance of DMS/DMSP-related genes exceeded 1 (Fig. 3a, b), suggesting that some bacteria may harbour more than one key gene in one or more DMS/DMSP metabolic pathways. We thus carried out co-occurrence analyses of key genes involved in DMS/DMSP metabolic pathways using MAGs assembled from these polar ocean metagenomes. Two hundred and fourteen microbial MAGs (> 80% completeness and < 2% potential contamination) belonging to 23 classes (Table S3) were recovered from these 60 polar metagenomes [69]. One hundred and forty-three MAGs affiliated with 15 classes including 70 families (Table S3) were found to contain at least one gene involved in DMS/DMSP cycling (Fig. 6a). Of these 143 MAGs, 63 MAGs had more than one key gene in the DMS/DMSP metabolic pathways. Overall, at the gene level, these MAGs had 13 different genes (as indicated by the nodes) and 28 co-occurrence combinations (as indicated by the edges, Fig. 6b). At the pathway level, the genes in these MAGs contributed to 7 different DMS/DMSP pathways with 12 co-occurrence combinations (Fig. 6c). According to the biological network analysis, the DMSP demethylation pathway (DmdA) and DMSP cleavage pathway (DddD) maintained the most frequent coexistence relationship (Fig. 6b, c), which also formed a close clustering relationship with genes responsible for the transformation between DMS and DMSO (Fig. 6b, c).

To uncover the co-occurrence of these genes in DMS/DMSP metabolism, we carried out a comprehensive co-occurrence network analysis of all microbial genomes in the IMG/M database, which contained genomes of 10285 isolates, 2120 Single cell Amplified Genomes (SAGs) and 5267 MAGs. At the time of the analysis (March 9, 2021), IMG/M included 2428 genomes, of which 412 genomes had more than one gene involved in DMS/DMSP metabolism (Table S9). At the gene level, these combinations yielded 50 one-to-one gene configuration modes (Fig. 6d), with DddP being the most frequent enzyme present in these genome-sequenced microbial strains, while DddL being the most connected gene coexisting with other genes involved in DMS/DMSP metabolism. At the pathway level, 14 different pathway co-occurrence patterns were observed (Fig. 6e). Interestingly, strong co-existence clustering among various DMSP-degradation pathways were observed in both MAGs from polar oceans and microbial genomes from the IMG/M, suggesting marine microbes likely employ multiple routes for DMSP catabolism. However, DMSP cleavage pathway and DMSP biosynthesis pathway showed stronger connection in microbial genomes from the IMG/M than MAGs from polar oceans metagenomes.

## Discussion

**Fig. 7:**
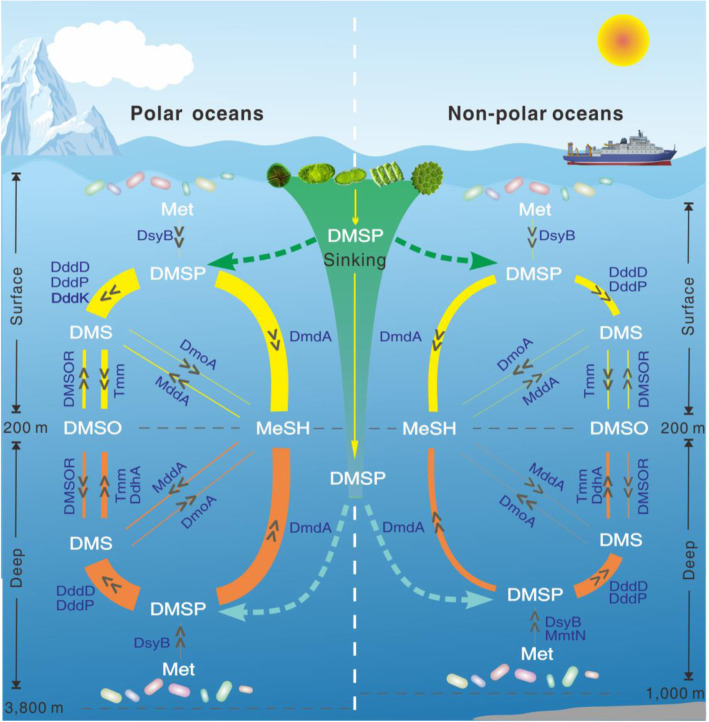
The conceptual diagram of bacterial DMS/DMSP metabolism in polar and non-polar oceans based on the analysis of the relative abundance of the potential genes involved in DMS/DMSP cycles. The thickness of the edge represents the relative abundance of the potential genes in each pathway. The arrowheads indicate the flow directions of organic sulfur compounds. Potential genes contributing more than 20% of the total relative abundance in each pathway are shown

Here, we investigated bacteria mediated DMS/DMSP cycling in 60 seawater metagenomes and 214 MAGs obtained from polar oceans and compared them with metagenomes and metatranscriptomes from the *Tara* Ocean datasets. The relative abundance and phylogenetic analyses of these potential genes involved in DMS/DMSP cycling in polar oceans suggested that there appears to be an intense and integrated DMS/DMSP cycle in polar oceans (Fig. 7). DmdA, DddD, DddP DddK, and Tmm appear to be the dominant genes involved in DMS/DMSP cycling, and Alpha- and Gamma-proteobacteria made the largest contributions. Globally, the geographic distribution of DMS/DMSP cycling was significantly influenced by water depth, which may be due to the differences in microbial assemblages caused by environmental selections. Furthermore, the coexistence of DMS/DMSP-related proteins in marine bacterial genomes was not a rare trait in polar oceans.

Met is the sulfocompound for the initiation of DMSP biosynthesis [34]. Given the presence of a low abundance of bacterial DMSP biosynthesis genes, DMSP in polar and non-polar oceans may largely be produced by phytoplankton in surface waters [27, 48, 58, 98, 99], which can then be transported to the deep ocean [100] through sinking particles.

Based on our analysis of the relative abundance of potential genes, a large proportion of DMSP may act as intermediates, while most of the sulfur from Met may ultimately be channelled into the production of DMS and especially MeSH. Considerable MeSH may thus accumulate in the polar oceans, which certainly warrants further investigation by measuring its in situ concentration in these polar environments. Our hypothesis is indeed supported by the high abundance and active transcription of DmdA in situ in metatranscriptomic samples (Fig. 3). Thus, the produced MeSH may provide a substantial budget for other physiological processes, such as MeSH oxidation to hydrogen sulfide by the MeSH oxidase (MTO) enzyme [74]. MTO was found to be abundant and widely distributed in both metagenomic and especially in metatranscriptomic samples in this study (Table S10).

Similarly, the relative abundance of Tmm and DMSOR in polar oceans suggested that the production DMS and DMSO were likely unbalanced, which may result in DMSO accumulation. Indeed, high concentrations of DMSO have been detected in both polar oceans waters and sea ice [25, 27, 47, 101], where they may act as cryoprotectants, osmoregulants, or cellular anti-oxidants in bacteria to cope with the extreme environments of the polar regions [102]. Besides, Tmm is also responsible for TMA oxidation to trimethylamine *N*-oxide (TMAO) [20] and the Tmm-mediated DMS oxidation to DMSO is a methylamine-dependent process [32], which suggests the presence of an inter-connected nitrogen-sulfur cycle through Tmm-mediated DMS oxidation.

Overall, the relative abundance of genes involved in DMS/DMSP cycling in polar oceans appears to be higher than that in non-polar oceans (Fig. 6). Interestingly, this corroborates with the fact that higher concentrations of DMS/DMSP were recorded at poles (Table 2) and turnover of DMS/DMSP at poles also appeared faster [26, 27] according to previous studies. Our results suggested that the dissimilarity of biogeographic traits of DMS/DMSP cycling was barely affected by dispersal limitation [103]. Instead, the similarities of environment conditions (i.e., illumination, temperature and salinity) at the same water layers may play a leading role [104]. The biogeographic traits tended to be more similarity in polar oceans which is consistent with bipolar distribution of marine bacteria [105, 106]. It is intriguing that biogeographic pattern of genes involved in DMS/DMSP cycling appears more similar in deep waters than surface waters. This may be due to the long-term stability and connectivity of deep waters [105]. However, there is still divergence between polar and non-polar surface waters, where the microbial communities suffered from short-term changing environmental conditions (e.g., changes in illumination and weather), consistent with the ecological theory that states ‘Everything is everywhere but the environment selects’ [107]. Future work on standing concentrations and turnover rates of these organic sulfurs and their response to environmental changes may shed new light on our understanding of their cycling in a changing climate.

## Conclusions

Overall, this study provides a global overview of the biogeographic traits of known bacterial genes involved in DMS/DMSP cycling from the Arctic and Antarctic oceans, laying a solid foundation for further studies of DMS/DMSP cycling in polar ocean microbiome at the enzymatic, metabolic, and processual levels.

## Supplementary Information

**Additional file 1: Figure S1**. Analysis of conserved amino acid residues involved in substrate binding and catalysis of DddK (a), DddQ (b), DddY (c), Tmm (d), DddP (e), DmdA (f) and DMSOR (g) retrieved from polar metagenomic samples. **Figure S2**. Maximum likelihood trees of the predicted hits of the top five most abundant genes (DddP, DddK, DddK, DmdA, Tmm) involved in DMSP/DMS cycling which were retrieved from the polar metagenomes, Tara metagenomes/metatranscriptomes datasets. **Figure S3**. Correlation between dissimilarity of DMS/DMSP related bacterial community and water depth in polar oceans.

**Additional file 2: Table S1**. Raw abundances of DMS/DMSP related genes in Arctic and Antarctic seawater samples. **Table S2**. A list of enzymes selected as outgroups for phylogenetic analyses. **Table S3**. Raw abundances and taxonomic composition of DMS/DMSP related genes in MAGs. **Table S4**. Raw abundances of DMS/DMSP related genes in *Tara* Ocean samples. **Table S5**. Raw abundances of DMS/DMSP related genes in metatranscriptomes. **Table S6**. The predicted hits with enzymatic activity. **Table S7**. Biplot scores for constraining variables. **Table S8**. Taxonomy composition of DMS/DMSP related genes in polar ocean samples. **Table S9**. Occurrence of DMS/DMSP related genes in genomes from IMG/M database. **Table S10**. Raw abundances of MTO in metagenomic and metatranscriptomic samples.

## Abbreviations

DMS: Dimethyl sulfide
DMSP: Dimethylsulfoniopropionate
MAGs: Metagenome assembled genomes
DMSO: Dimethylsulfoxide
MeSH: Methanethiol
TMA: Trimethylamine
TMAO: Trimethylamine *N*-oxide
Met: Methionine
MTHB: Methylthiohydroxybutryate
MMPA: Methylmercaptopropionate
DMSOP: Dimethylsulfoxonium propionate
BLAST: Basic local alignment search tool
GTDB: Genome taxonomy database
NCBI: National center for biotechnology information
HMM: Hidden markov models
PCoA: Principal coordinates analysis
RDA: Redundancy analysis
HNLC: High nutrient, low chlorophyll
