# Correction to: Biogeographic traits of dimethyl sulfide and dimethylsulfoniopropionate cycling in polar oceans

**DOI:** 10.1186/s40168-021-01182-y

**Published:** 2021-11-09

**Authors:** Zhao-Jie Teng, Qi-Long Qin, Weipeng Zhang, Jian Li, Hui-Hui Fu, Peng Wang, Musheng Lan, Guangfu Luo, Jianfeng He, Andrew McMinn, Min Wang, Xiu-Lan Chen, Yu-Zhong Zhang, Yin Chen, Chun-Yang Li

**Affiliations:** 1 State Key Laboratory of Microbial Technology, Marine Biotechnology Research Center, Shandong University, Qingdao, 266237 China; 2 College of Marine Life Sciences, Institute for Advanced Ocean Study, Ocean University of China, Qingdao, 266003 China; 3 Laboratory for Marine Biology and Biotechnology, Pilot National Laboratory for Marine Science and Technology (Qingdao), Qingdao, 266373 China; 4 The Key Laboratory for Polar Science MNR, Polar Research Institute of China, Shanghai, 200136 China; 5 Institute for Marine and Antarctic Studies, University of Tasmania, Hobart, Tasmania Australia; 6 School of Life Sciences, University of Warwick, Coventry, UK

**Correction to: Microbiome 9, 207 (2021).**

**https://doi.org/10.1186/s40168-021-01153-3**

Following the publication of the original article [1], the author reported that there is an error in one of the authors name. The name of the 8th co-author of this article should be “Guangfu Luo”, not “Guangfu Lu”.

The original article has been updated.
