# Supplementary material for: Biogeographic traits of dimethyl sulfide and dimethylsulfoniopropionate cycling in polar oceans
Source: Microbiome. 2021 Oct 16;9:207. doi: 10.1186/s40168-021-01153-3 (PMC8520302; doi:10.1186/s40168-021-01153-3)
Supplement: Supplementary file 2 — Additional file 1: Figure S1. Analysis of conserved amino acid residues involved in substrate binding and catalysis of DddK (a), DddQ (b), DddY (c), Tmm (d), DddP (e), DmdA (f) and DMSOR (g) retrieved from polar metagenomic samples. Figure S2. Maximum likelihood trees of the predicted hits of the top five most abundant genes (DddP, DddK, DddK, DmdA, Tmm) involved in DMSP/DMS cycling which were retrieved from the polar metagenomes, Tara metagenomes/metatranscriptomes datasets. Figure S3. Correlation between dissimilarity of DMS/DMSP related bacterial community and water depth in polar oceans. [file 40168_2021_1153_MOESM2_ESM.docx]

**
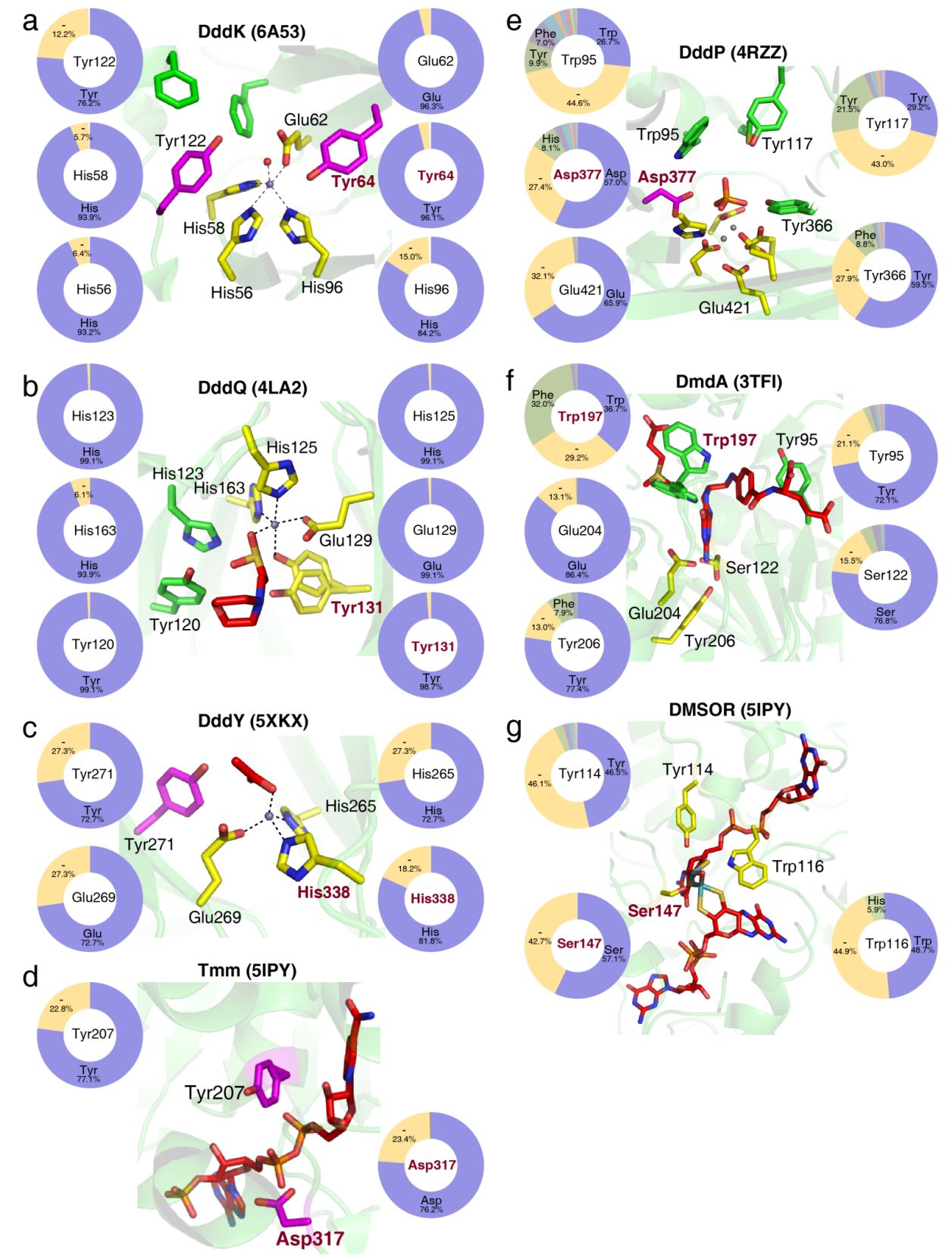
**

**Figure S1. Analysis of conserved amino acid residues involved in substrate binding and catalysis of DddK (a), DddQ (b), DddY (c), Tmm (d), DddP (e), DmdA (f) and DMSOR (g) retrieved from polar metagenomic samples.** The PDB code of each protein was shown in the bracket. The amino acid residues suffixed with numbers represent the conserved sites of each protein. The pie charts in each panel show the amino acid compositions of corresponding conserved sites in all predicted hits from polar metagenomes. The key conserved residues involved in catalysis of each protein is shown in red bold text. “-” indicates the absence of corresponding amino acid residue from environmental hits owing to their short sequence length. Only amino acid residues > 5% in each position are shown.


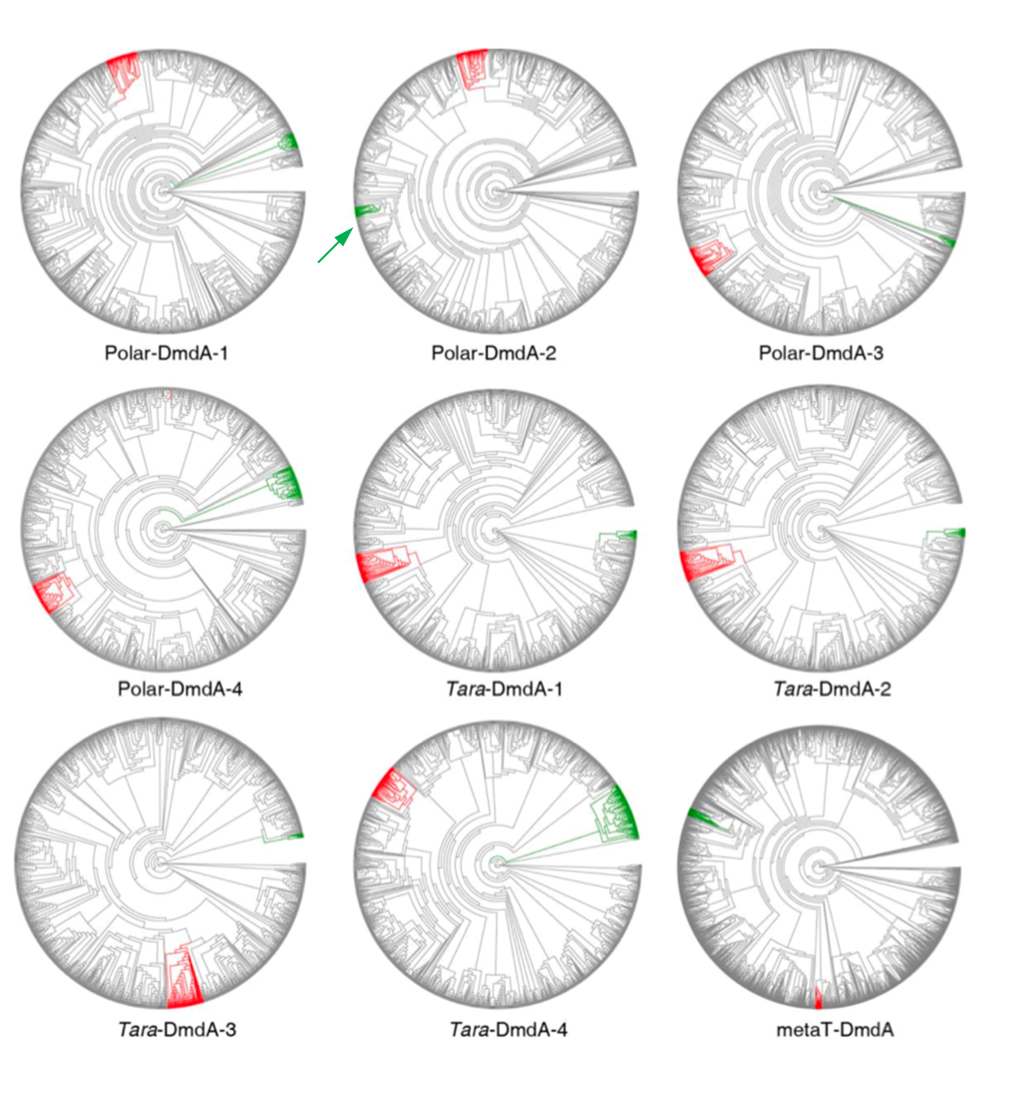

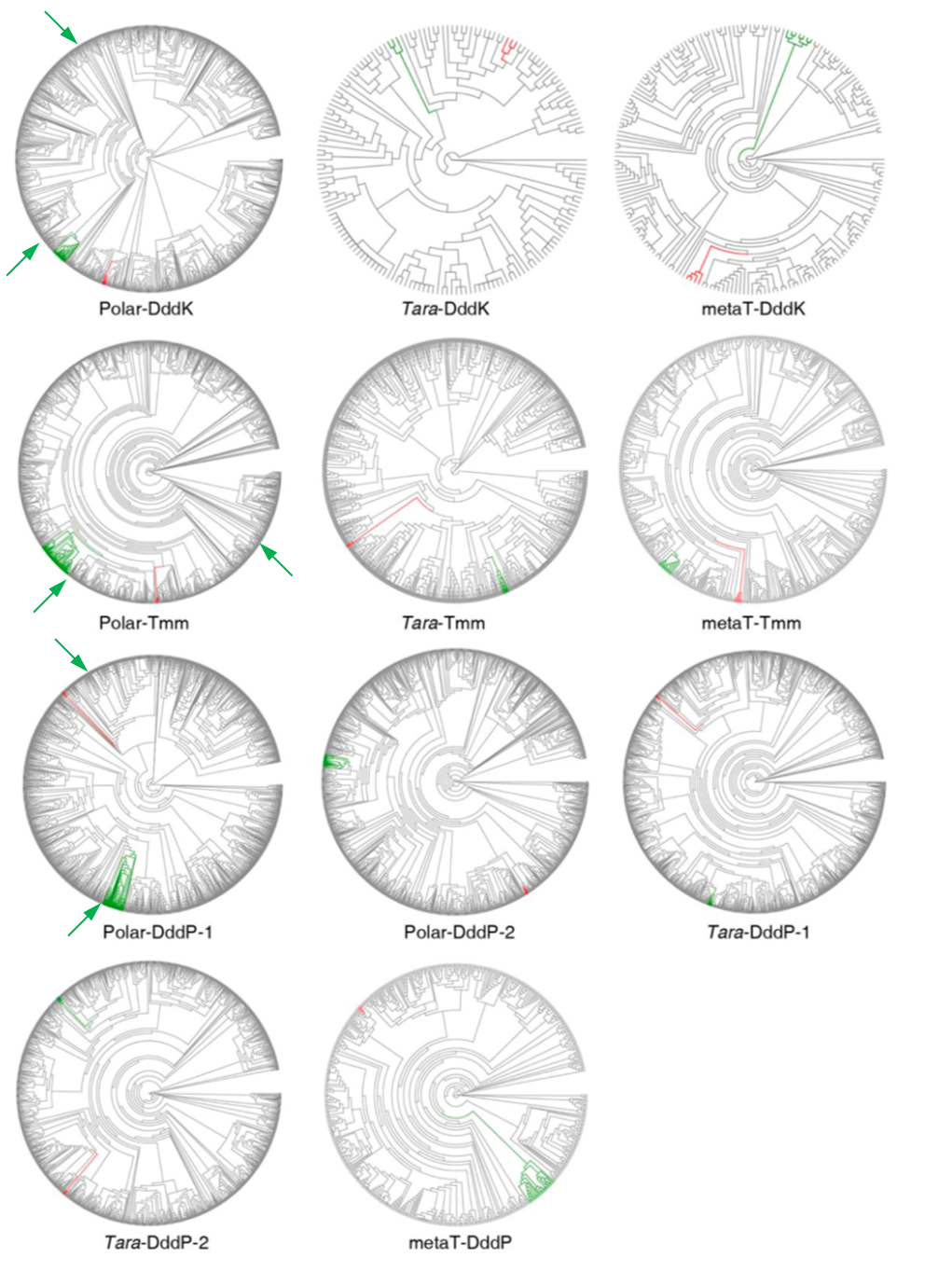


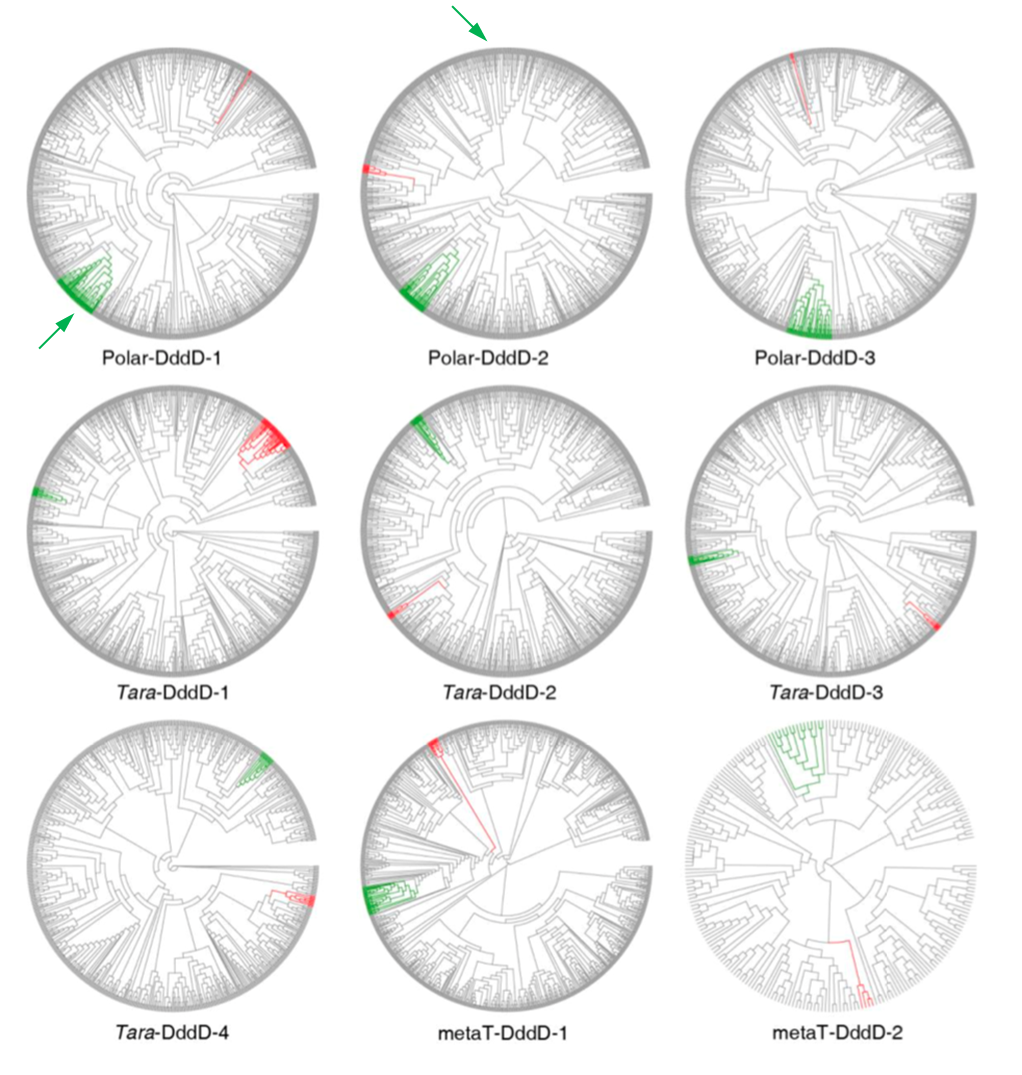


**Figure S2. Maximum likelihood trees of the predicted hits of the top five most abundant genes (DddP, DddK, DddK, DmdA, Tmm) involved in DMSP/DMS cycling which were retrieved from the polar metagenomes, Tara metagenomes/metatranscriptomes datasets.** For DddP, DmdA and DddD, environmental hits were split into several subsets with no more than 2000 sequences for multiple sequence alignment at one time using MUSCLE. The split subsets were suffixed with numbers (1-4). The clusters containing ratified enzymes are shown in green. Those shown in red were environmental sequences with other/unknown functions which were excluded for further taxonomic and statistical analyses. Functionally characterized environmental sequences (see Table S6) were highlighted by green arrows.

**
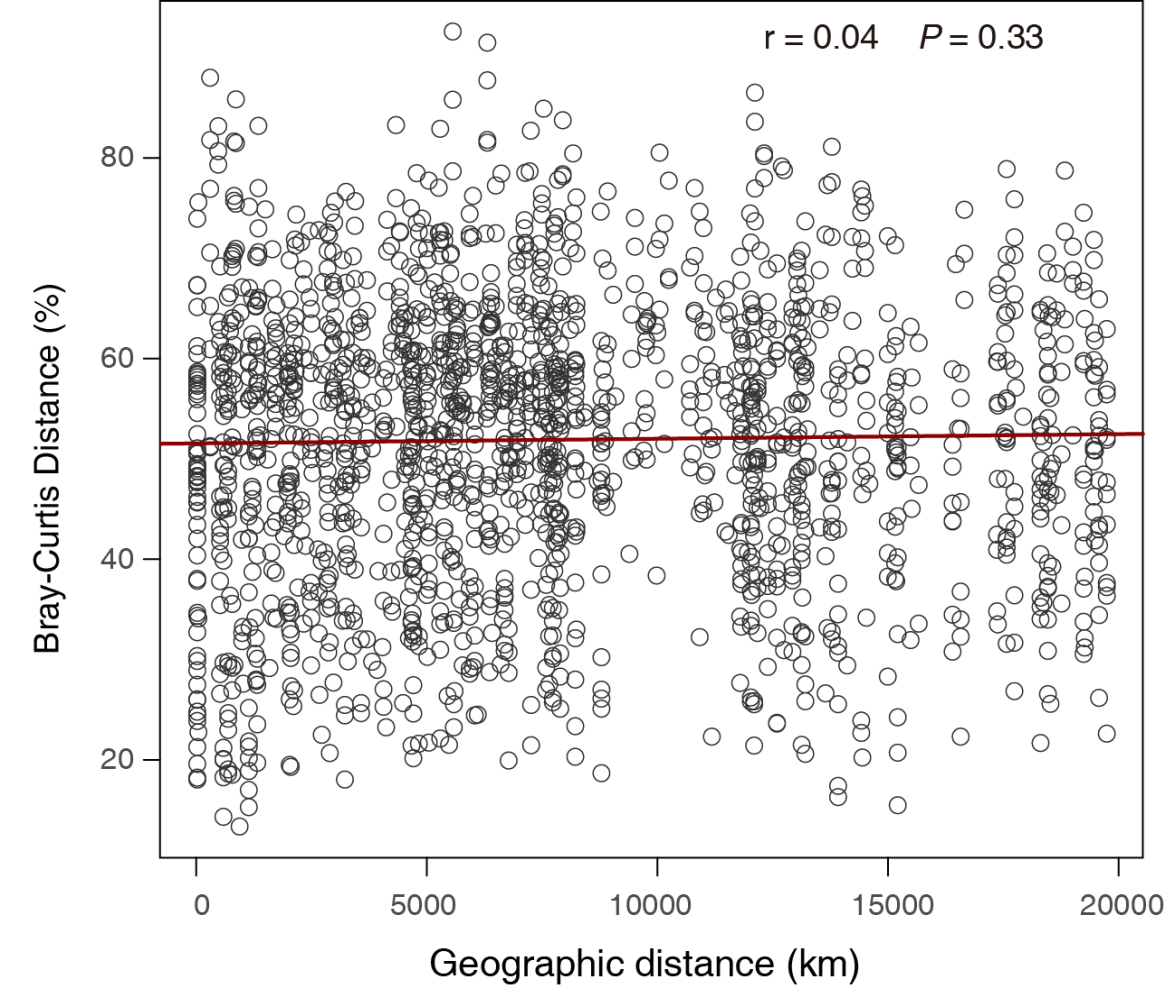
**

**Figure S3. Correlation between dissimilarity of DMS/DMSP related bacterial community and water depth in polar oceans.** The Bray-Curtis dissimilarity index was used. The correlation coefficients (r) and Spearman’s correlation *P* value (*P*) were indicated.
